# Chloride intracellular channel 1 functions in endothelial cell growth and migration

**DOI:** 10.1186/2040-2384-2-23

**Published:** 2010-11-01

**Authors:** Jennifer J Tung, Jan Kitajewski

**Affiliations:** 1 Department of Obstetrics/Gynecology, Herbert Irving Comprehensive Cancer Center, Columbia University Medical Center, 1130 St. Nicholas Ave, 926, New York, NY 10032, USA; 2 Department of Pathology, Herbert Irving Comprehensive Cancer Center, Columbia University Medical Center, 1130 St. Nicholas Ave, 926, New York, NY 10032, USA

## Abstract

**Background:**

Little is known about the role of CLIC1 in endothelium. These studies investigate CLIC1 as a regulator of angiogenesis by *in vitro *techniques that mimic individual steps in the angiogenic process.

**Methods:**

Using shRNA against *clic1*, we determined the role of CLIC1 in primary human endothelial cell behavior.

**Results:**

Here, we report that reduced CLIC1 expression caused a reduction in endothelial migration, cell growth, branching morphogenesis, capillary-like network formation, and capillary-like sprouting. FACS analysis showed that CLIC1 plays a role in regulating the cell surface expression of various integrins that function in angiogenesis including β1 and α3 subunits, as well as αVβ3 and αVβ5.

**Conclusions:**

Together, these results indicate that CLIC1 is required for multiple steps of *in vitro *angiogenesis and plays a role in regulating integrin cell surface expression.

## Background

The chloride intracellular channel (CLIC) gene family consists of seven distinct paralogues (p64 and CLIC1-6) and constitutes a unique class of mammalian channel proteins that exist as both cytoplasm-soluble proteins and membrane-bound channels [1]. CLICs are structurally related to the glutathione S-transferase (GST) superfamily and are defined by an approximately 240 conserved amino acid sequence at the C-terminus [2]. Most of the distinct CLIC proteins are shown to form channels in artificial bilayers [3-7], but their selectivity for chloride as channels is still under contention [8,9]. CLICs and their homologues are highly conserved among both vertebrates and invertebrates [10,11].

Since their discovery, members of the CLIC family have been implicated in such diverse biological processes as apoptosis [12], differentiation [12,13], cell cycle regulation[1], and cell migration [9] in a variety of different cell types. In separate studies, CLIC4 is found to promote endothelial proliferation and morphogenesis [14] and to function in mouse retinal angiogenesis [15]. The current model for the angiogenic function of CLIC4 involves CLIC4 channel activity in the acidification of vesicles [15], a process that may be linked to lumen formation or tubulogenesis [16]. The Hobert group also demonstrates the requirement of *C. elegans *CLIC4 orthologue EXC-4 expression in preventing cystic disruption of an expanding *C. elegans *excretory canal and defines a role for EXC-4 in maintaining proper excretory canal lumen size [17]. A chimeric construct expressing human CLIC1 with the putative transmembrane domain (PTM) of *exc4 *is able to rescue the cystic disruption phenotype of the excretory canal in *exc4 *null mutants, suggesting that CLIC4 and CLIC1 may have overlapping functions [10].

To date, six CLIC genes (CLIC 1-6) are identified in mice and humans, and CLIC1 and CLIC4 are reported to be strongly expressed in endothelial cells [17-19]. As CLIC4 is linked to the process of angiogenesis and lumen formation within endothelial cells [15,20], interest in the possibility that other CLICs are involved in angiogenesis has grown. Structural studies indicate that oxidized CLIC1 forms dimers in artificial bilayers and vesicles with the PTM located near the N-terminus [4,21]. It is also suggested that CLIC1 activity is dependent on pH [22]. Studies localize CLIC1 to the nuclear membrane and it is suggested that CLIC1 can regulate the cell cycle of CHO-K1 cells [1]. CLIC1 is almost ubiquitously expressed in human and mouse adult and fetal tissue [1] and is shown to be F-actin regulated, suggesting that it could function in solute transport, during any number of stages in the cell cycle, or during cell migration [9]. In several columnar epithelia tissue samples, including but not limited to the renal proximal tubes, small intestine, colon, and airways, CLIC1 is found to be expressed in the apical domains suggesting a role in apical membrane recycling [18]. The same study also finds that CLIC1 subcellular distribution is polarized in an apical fashion in human colon cancer cells while another study finds it localized to intracellular vesicles in renal proximal tubule cells [23]. Since the process of angiogenesis is known to involve endothelial cytoskeletal reorganization, apical-basal polarization, and proliferation [24,25], these studies suggest CLIC1 may function in endothelial morphogenesis by influencing some or all of these cellular and subcellular processes.

Most recently, the Breit group generated a CLIC1 knockout mouse and report platelet dysfunction as well as inhibited clotting in CLIC1 nullizygous mice [26]. There are no other gross phenotypes reported in the CLIC1 nullizygous mice. Given the previously defined roles of CLIC4 in angiogenesis, the suggestion of functional redundancies between CLIC4 and CLIC1, and the implications of CLIC1 involvement in cytoskeletal organization and apical membrane recycling, we now seek to define the role of CLIC1 in endothelial cell behavior and angiogenesis.

Here, we demonstrate the importance of CLIC1 expression in multiple steps of *in vitro *angiogenesis as well as elucidating a role for CLIC1 in regulating integrin cell surface expression. We show that with reduced CLIC1 expression there is reduced endothelial migration, cell growth, branching morphogenesis, capillary-like network formation, and capillary-like sprouting. CLIC1 also plays a role in regulating the cell surface expression of various integrins important in angiogenesis, including αVβ3 and αVβ5 and subunits β1 and α3.

## Methods

### Antibodies

Primary polyclonal rabbit anti-human CLIC1 (B121) antibody was a gift from Mark Berryman at Ohio University College of Osteopathic Medicine (Athens, OH) [27]. Primary polyclonal rabbit anti-human CLIC4 antibody was purchased from Abcam Inc. (Cambridge, MA) while primary monoclonal mouse anti-α-tubulin antibody was purchased from Sigma-Aldrich (St. Louis, MO). Primary monoclonal mouse anti-human antibodies for integrin subunit chains α2, β1, and α3 were purchased from BD Biosciences (San Jose, CA) and primary monoclonal mouse anti-human antibodies for integrins αVβ3 and αVβ5 were purchased from Millipore (Billerica, MA). Primary monoclonal mouse anti-human CD31 antibody was purchased from Dako (Carpinteria, CA). Secondary goat anti-rabbit and goat anti-mouse horseradish peroxidase (HRP)-conjugated antibodies were purchased from Sigma (St. Louis, MO). Secondary goat anti-mouse allophycocyanin (APC)-conjugated AffiniPure IgG antibody was obtained from Jackson ImmunoResearch Laboratories, Inc. (West Grove, PA).

### Cell culture

Human umbilical venous endothelial cells (HUVEC) were isolated from human umbilical veins as described previously [28]. HUVEC were cultured on dishes coated with Type I rat tail collagen (VWR, West Chester, PA) in EGM-2 BulletKit medium (Lonza, Basel, Switzerland) without hydrocortisone unless otherwise noted. Detroit 551 fibroblasts and 293T cells (ATCC, Manassas, VA) were cultured in Eagle's Minimum Essential Medium (ATCC, Manassas, VA) and Iscove's Modified Dulbecco's Medium (Invitrogen, Carlsbad, CA), respectively. Both media were supplemented with 10% fetal bovine serum and 1× Pen-Strep (Invitrogen, Carlsbad, CA). All cells were maintained under standard humidified incubator conditions at 37°C and 5% CO_2_.

### CLIC1 gene silencing

A human *clic1 *shRNA-containing construct in lentiviral vector pLKO.1-puro (Sigma-Aldrich, St. Louis, MO) was used to provide *clic1 *knockdown in HUVEC, which was confirmed by immunoblotting. The *clic1*-targetting shRNA possessed the target sequence of 5'-CCTGTTGCCAAAGTTACACAT-3'. Lentiviral vector pLKO.1-puro expressing scrambled shRNA that does not target any known human genes was used as the control (Sigma-Aldrich, St, Louis, MO). pLKO.1-puro plasmids were used for lentivirus-mediated stable expression of *clic1 *shRNA or scrambled shRNA in HUVEC. To generate lentiviral particles for stable infection, 2.5 × 10^6 ^293T cells were seeded on a 10 cm tissue culture dish and transfected with lentivirus-packaging components pVSVG (3 μg), pMDLg/pPRE (5 μg), and pRSV-Rev (2.5 μg) along with 10 μg appropriate pLKO.1-puro plasmid. 293T-generated lentivirus-containing supernatants were then collected at 48 and 56 h post-transfection, filtered through a 0.45 μm syringe filter, and immediately added to low-passage HUVEC seeded at 1 × 10^6 ^cells per 10 cm collagen-coated dish for stable infection. 48 h after the last infection, pLKO.1-puro-expressing HUVEC were selected with puromycin at 3 μg/mL for 72 h and maintained with puromycin in EGM-2 at 1.5 μg/mL.

### Immunoblotting

HUVEC protein lysates were prepared in TENT lysis buffer (50 mM Tris pH 8.0, 2 mM EDTA, 150 mM NaCl, and 1% Triton X-100) containing Protease Inhibitor Cocktail Set IV (EMD Chemicals, Inc., Gibbstown, NJ) prepared according to the manufacturer's protocol. Lysates were boiled for 5 min after addition of SDS and β-mercaptoethanol-containing sample buffer. Protein concentrations were determined using the Bradford protein assay (Bio-Rad Laboratories, Hercules, CA) according to the manufacturer's protocol, and sample volumes were adjusted to equivalent concentrations for equal protein loading into SDS-PAGE. Protein was then electroblotted onto nitrocellulose membrane and blocking occured in 5% milk dissolved in PBST (1× PBS with 0.2× Tween20). Incubation of primary antibody (1:250 for CLIC1; 1:250 for CLIC4; or 1:5000 for α-tubulin) was done in 2.5% milk dissolved in PBST, and incubation of secondary antibody (1:5000 for both HRP-conjugated goat anti-rabbit and goat anti-mouse) occurred in 2.5% milk in PBST. Protein bands were visualized using Enhanced Chemiluminescence (GE Healthcare Bio-Sciences Corp., Piscataway, NJ) according to the manufacturer's protocol.

### Cell viability and cell growth assays

HUVEC were seeded at 3 × 10^4 ^cells/well of a 24-well plate and cultured in either serum free medium (SFM) alone or SFM with 20 ng/mL epidermal growth factor (EGF) for 48 h for cell viability assays (Invitrogen, Carlsbad, CA). Cell numbers after 48 h were quantified using Cell Counting Kit-8 WST-8 (Dojindo Molecular Technologies, Gaithersburg, MD) according to the manufacturer's protocol. Similarly for cell growth assays, HUVEC were seeded at 1 × 10^4 ^cells/well of a 24-well plate and cultured in SFM with 20 ng/mL EGF and 20 ng/mL recombinant human vascular endothelial growth factor A (rhVEGF) for 96 h (Invitrogen, Carlsbad, CA). Again, cell numbers were scored using Cell Counting Kit-8 WST-8, and a calibration curve was generated following Dojindo's protocol (Gaithersburg, MD). Assays were performed in triplicate and replicated at least five times.

### Migration "scratch" analysis and TScratch quantification

As previously described, HUVEC were seeded to confluence at 1 × 10^6 ^cells/well of a 6-well plate and cultured in EGM2 medium [14]. 24 h post-seeding, cell monolayers were bisected along the diameter of each well with a 200 μL pipette tip, creating an open "scratch" or "wound" that was clear of cells. The dislodged cells were removed by three washes with 1× PBS, EGM-2 medium was replaced, and cells were incubated under standard conditions. Migration into the open area was documented at 0, 3, 6, 9, and 12 h post-scratching. Quantification was done using TScratch software and performed according to the creator's protocol [29]. Experiments were done in triplicate and repeated at least five times.

### Flow cytometry

1.5 × 10^5 ^HUVEC were incubated at 4°C on a rotator for 1 h with primary antibody (1:50 for each of α2, β1, α3, αVβ3, αVβ5, CD31, and without primary for negative controls) in PCN (0.1% NaN_3_, 0.001 M MgCl_2_, 0.5% FBS in 1× PBS). Cells were incubated with secondary antibody (1:100 for APC-conjugated goat anti-mouse in PCN) on a rotator for 45 min at 4°C and kept on ice overnight. The BD FACSCalibur was used to perform flow cytometry according to the manufacturer's protocol and data analysis was performed using CD CellQuest Pro software (San Jose, CA). Experiments were repeated at least three times.

### Network formation assay and quantification

HUVEC were cultured at standard conditions as a monolayer between two layers of porcine collagen gel (Wako USA, Richmond, VA) as previously described for the network formation assay [14,30]. Briefly, HUVEC were seeded between two layers of porcine collagen gel at 1 × 10^5 ^cells/well of a 24-well plate. Gels were cultured in SFM supplemented with 20 ng/mL EGF and 20 ng/mL rhVEGF for 96 h. For quantification, MTT (Dojindo Molecular Technologies, Gaithersburg, MD) was applied to gels for 3 h at the termination of the assay as previously described [14]. Image-Pro Plus software (Media Cybernetics, Bethesda, MD) was then used to calculate the surface area occupied by MTT-treated HUVEC. Branchpoints were tabulated at the termination of the assay with one branchpoint considered to be any intersection of two or more cords. Experiments were performed in triplicate and repeated at least five times.

### Capillary-like sprouting fibrin bead assay and quantification

The capillary-like sprouting assay was performed as previously described [31] with two additional modifications [14]. Briefly, primary HUVEC and Detroit 551 fibroblasts were exposed to M199 medium with 10% FBS and 1× Pen-Strep (Invitrogen, Carlsbad, CA). HUVEC were attached to dextran-coated Cytodex 3 beads (GE Healthcare Bio-Sciences Corp., Piscataway, NJ) at 400 HUVEC/bead and embedded at 250 beads/well of a 24-well plate in a fibrin clot. D551 fibroblasts were then seeded as a monolayer over the clot at 1.5 × 10^5 ^cells/well. Clots were cultured at standard conditions in EGM-2 medium and the assay was allowed to run for 11 days. Experiments were performed in triplicate and replicated at least three times.

### Statistics

Unless otherwise noted, independent two-tailed Student's t-tests were performed on all quantified data to determine significant differences. P values less than 0.05 were considered statistically significant, and equal variances were assumed.

### Ethics statement

No human or animal subjects were used in this study. The collection of HUVEC from umbilical cords was approved by Columbia University IRB-AAAE4646.

## Results

### Generation of endothelial cell lines with stable CLIC1 knockdown

**Figure 1:**
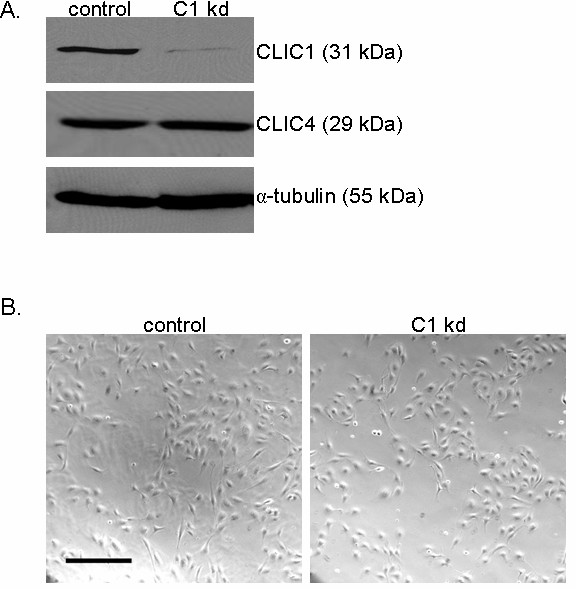
**Establishing human endothelial cell lines with CLIC1 knockdown**. A. Immunoblotting with polyclonal rabbit anti-CLIC1 antibody confirmed reduced CLIC1 protein expression in CLIC1 knockdown (C1 kd) endothelial cell lines. Immunoblotting with rabbit polyclonal anti-CLIC4 antibody verified that CLIC4 expression was not altered by *clic1*-targetting shRNA. Alpha-tubulin served as a loading control. B. Established HUVEC control and CLIC1 knockdown lines exhibited no gross morphological differences when cultured as a subconfluent monolayer on collagen-coated dishes. Scale bar, 30 μm.

Human umbilical venous endothelial cell (HUVEC) lines with stable CLIC1 knockdown expression were used to determine the function of CLIC1 in various steps of angiogenesis, modeled with *in vitro *assays. Endothelial cell lines were generated using lentiviral-based vectors carrying *clic1*-targeting shRNA. Immunoblotting for CLIC1 confirmed knockdown at the protein level with a reduced band around 31 kDa (Figure 1a) [27]. Immunoblotting for CLIC4 indicated that the *clic1 *shRNA did not target CLIC4 and that CLIC4 expression levels were not affected by CLIC1 knockdown. CLIC1 knockdown HUVEC phenotypes were compared to control endothelial cells stably expressing the lentivirus-based vector backbone carrying a scrambled shRNA insert that is not known to target any human genes (hereafter referred to as "control").

The cellular morphology of CLIC1 knockdown HUVEC was qualitatively assessed and we observed no major changes in morphology with respect to control (Figure 1b). For this analysis, the CLIC1 knockdown and control cell lines were seeded in equal numbers as a subconfluent monolayer on collagen-coated plates and photographed 48 h later. While no gross morphological changes were present, we noted that CLIC1 knockdown cells appeared less dense than control cells, indicating that CLIC1 knockdown may affect endothelial cell growth.

### CLIC1 knockdown inhibits endothelial cell growth

**Figure 2:**
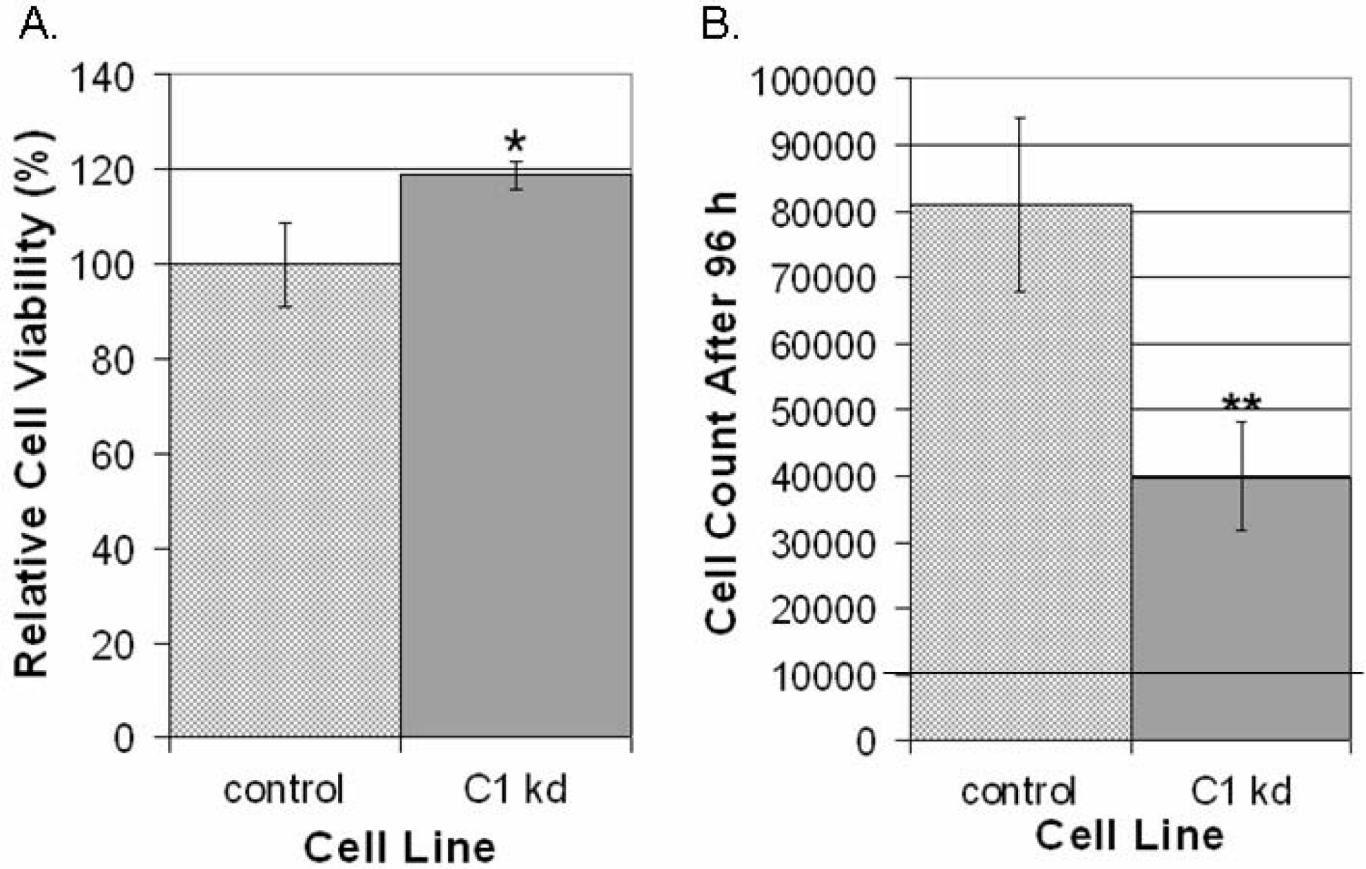
**CLIC1 knockdown has mild effects on viability and significantly inhibits endothelial cell growth**. A. CLIC1 knockdown mildly but significantly increased viability in endothelial cells 48 h post-seeding. Quantification of viability is shown as mean percentages relative to the respective starting number of cells with standard deviation. (*p < 0.05). B. Reduced CLIC1 expression drastically and significantly inhibited endothelial cell growth. The line at 10000 cells on the y-axis indicates the number of cells at the start of the assay. Quantification of cell growth is shown as the mean number of cells for each group with standard deviation. (**p < 0.001).

To determine if CLIC1 is involved in regulating endothelial cell viability or growth, we utilized WST-8 colorimetric cell counting assays to score cells under different conditions. Cell viability assays were performed on CLIC1 knockdown or control cells by seeding equal numbers of HUVEC on collagen-coated plates and scoring cells after being cultured for 48 h in serum-free medium (SFM) alone or SFM supplemented with the survival signal EGF. Cell counts in medium without survival factor EGF were then scored relative to cell counts of the same cell line in medium with EGF to produce a percentage of cells that remain viable in medium without EGF. In the absence of EGF, we found that CLIC1 knockdown cells displayed a modest but significant increase in HUVEC viability (p < 0.05), indicating that CLIC1 knockdown may play a limited role in regulating cell viability (Figure 2a).

To determine the effects of knocking down CLIC1 on endothelial cell growth, CLIC1 knockdown or control HUVEC were seeded in equal numbers on collagen coated plates and cultured in SFM supplemented with survival signal EGF and VEGF to induce endothelial cell growth. Cells were allowed to grow for 96 h, and cells were then scored using WST-8 colorimetric detection. In contrast to the effect on cell viability, we found that reduction of CLIC1 expression led to a pronounced and significant reduction of HUVEC cell growth (p < 0.001), indicating that CLIC1 is involved in regulating endothelial cell growth (Figure 2b).

### CLIC1 knockdown inhibits endothelial migration

**Figure 3:**
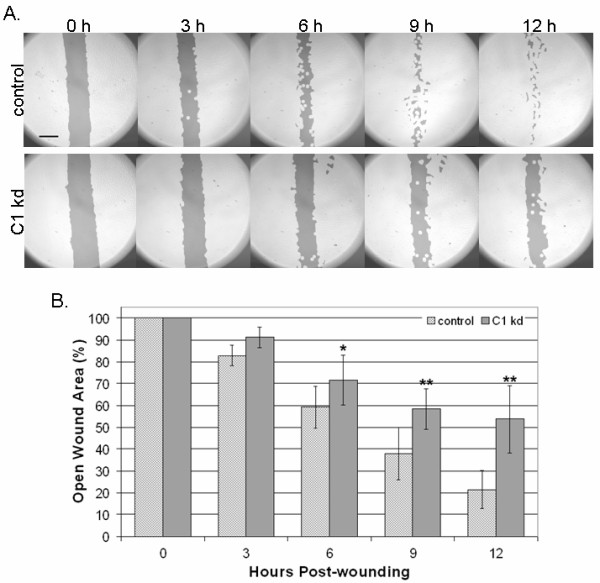
**Reduced CLIC1 expression inhibits endothelial migration**. A. CLIC1 knockdown endothelial lines exhibited inhibited directed migration in a scratch assay, noticeable as early as 3 h post-wounding. Photographs shown here have been analyzed using the TScratch software for quantifying open surface area. Lighter shaded areas indicate area occupied by endothelial cells while darker shaded areas indicate surface area not occupied by endothelial cells. Scale bar, 50 μm. B. Quantification using the TScratch software showed that there was significantly more open surface area in the CLIC1 knockdown group at and after 6 h post-wounding. Mean open surface area for each group is shown with standard deviation. (*p < 0.02; **p < 0.001).

Given the previously described associations of CLIC1 with cytoskeletal elements [9], we utilized a directed cell migration scratch assay to determine if CLIC1 knockdown affects directed endothelial cell motility. This assay involved creating an "open wound" across a confluent monolayer of HUVEC growing on collagen and documenting cell migration into the wounded area at various time points over a 12 h period. We found that CLIC1 knockdown resulted in reduced endothelial migration into the wounded area when assessed on a qualitative level (Figure 3a). Given that the CLIC1 knockdown migratory defects are qualitatively noticeable as early as 3 h post-wounding and the migration assay terminated only 12 h post-wounding, it was unlikely that any CLIC1 knockdown effects on endothelial survival or proliferation affected migration assay outcomes.

To quantify the extent to which reduced CLIC1 expression was inhibiting directed endothelial cell migration, TScratch software was used for quantifying open surface area [29]. Quantification of data collected from six separate experiments confirmed that CLIC1 knockdown cells occupy significantly less surface area at 6 (p < 0.02), 9 (p < 0.001), and 12 h (p < 0.001) post-wounding (Figure 3b), indicating that CLIC1 knockdown significantly reduced directed endothelial cell migration as early as 6 h post-wounding. Thus, inhibiting CLIC1 expression reduces directed endothelial cell migration.

### CLIC1 influences expression of select integrins in endothelial cells

To explore the regulatory role of CLIC1 in endothelial migration further, we analyzed the effects of CLIC1 knockdown on various integrins by flow cytometry. For this assessment, CLIC1 knockdown and control HUVEC were cultured as subconfluent monolayers on either Type I collagen-coated or fibronectin-coated plates and incubated with primary antibodies for various integrins. A secondary APC-conjugated antibody was then used to enable flow cytometry. Integrins examined include α2, β1, α3, αVβ3, and αVβ5. Endothelial marker CD31 served as a positive control while the absence of primary antibody served as a negative control. In addition to being important for cell attachment and migration on specific extracellular matrices, each of the integrins examined have been reported to affect the angiogenic process [32-36].

**Figure 4:**
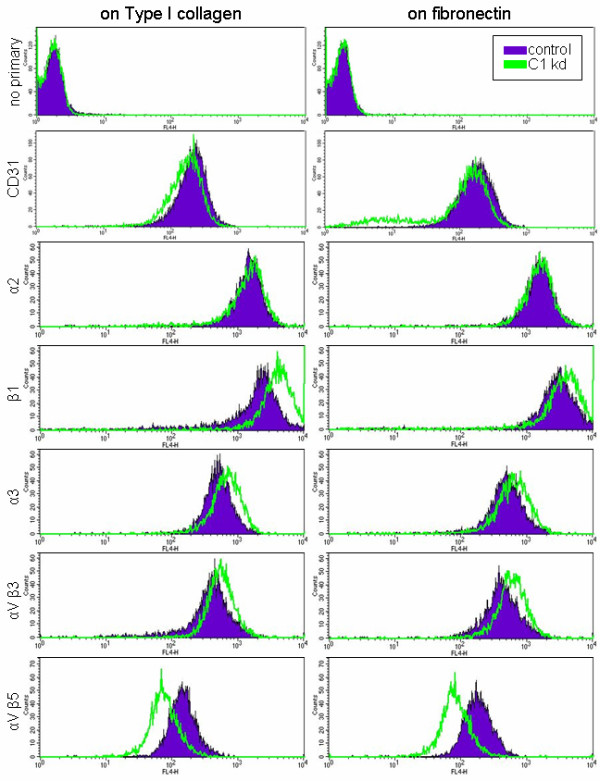
**CLIC1 expression plays a role in the expression of various integrins**. Histograms from a typical flow experiment indicate that HUVEC with CLIC1 knockdown (green) had increased β1, α3, and αVβ3 expression and decreased αVβ5 expression while α2 expression remained generally unchanged. Trends persisted despite being cultured on collagen or fibronectin extracellular matrix components. A downward shift in positive control endothelial marker CD31 was typical with CLIC1 knockdown cells.

Expression analysis revealed the same general expression patterns for HUVEC grown either on collagen-coated plates (Figure 4, left panels) or HUVEC grown on fibronectin-coated plates (Figure 4, right panels). The expression assays were done three times and results from a typical flow experiment are depicted in Figure 4. A slight downward shift in CD31 was found to be typical of CLIC knockdown cells. Analysis from both plate types indicated that HUVEC with CLIC1 knockdown had increased cell surface expression of integrin subunits β1 and α3 while α2 remained mostly unaffected (Figure 4). Integrin αVβ3 surface expression was also increased in CLIC1 knockdown endothelial cells while αVβ5 expression was decreased on both extracellular matrix plate types. These results suggest that CLIC1 may be moderating endothelial migration through regulation of integrin expression at the cell surface.

### CLIC1 plays a role in capillary-like network formation

**Figure 5:**
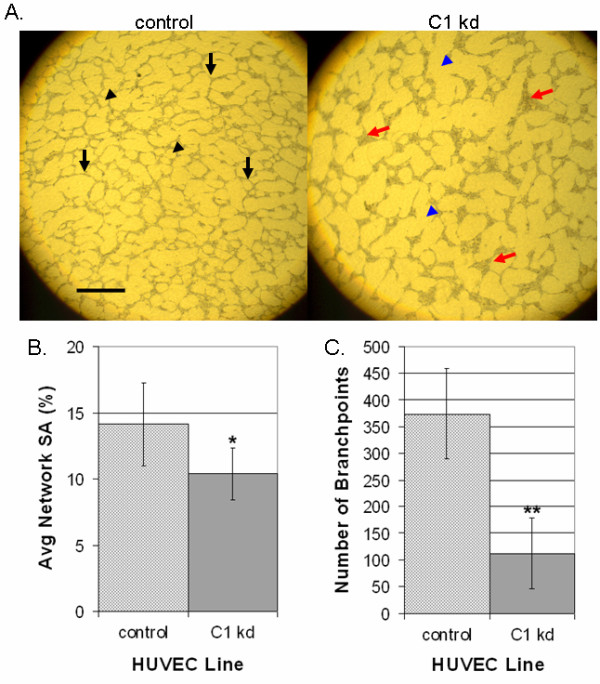
**Knocked down CLIC1 expression inhibits capillary-like network formation and branching morphogenesis**. A. Microscopy of control or CLIC1 knockdown lines in a collagen network formation assay indicated decreased network formation and defective branching morphogenesis in cells with CLIC1 knockdown. In addition to having less dense network patterning (exemplified by blue arrowheads), cell aggregations were found where normal branchpoints should have been (exemplified by red arrows). Black arrowheads indicate regularly spaced network patterning, and black arrows indicate normal branchpoints. Scale bar, 50 μm. B. Quantification of surface area occupied by endothelial cells showed a significant decrease in surface area occupation by cells with CLIC1 knockdown. Mean surface area occupied by endothelial cells for each cell line is shown with standard deviation. (*p < 0.01). C. Quantification of branchpoint numbers revealed a significant decrease in the number of branchpoint formations in cells with CLIC1 knockdown. One branchpoint was considered any focal intersection of two or more cords. The mean number of branchpoints for each cell line is shown with standard deviation. (**p < 0.001).

We next determined the effect of CLIC1 knockdown on endothelial cell morphogenesis by assessing the ability of HUVEC to organize into capillary-like networks. To do this, CLIC1 knockdown or control HUVEC were seeded in equal numbers between two layers of porcine collagen gel and cultured in SFM supplemented with VEGF and EGF for 96 h. Qualitative assessment of the results indicated a reduction in cord, network, and branch formations in CLIC1 knockdown relative to control (Figure 5a). Network formation from cells with reduced CLIC1 expression was noticeably less dense when compared to control cells as indicated by more prevalent open surface area (indicated by blue arrowheads, compared to control black arrowheads). In addition to these defects, we found dramatic cell aggregations at branchpoints when CLIC1 was knocked down, where one branchpoint was considered any intersection of two or more cords (indicated by red arrows, compared to control black arrows). This phenotype could have been due to the migratory defects established earlier. As indicated in a previous study assessing CLIC4 function, these cell aggregations are not due to proliferative defects [14].

Quantification of these results revealed that there is a significant reduction in surface area coverage by CLIC1 knockdown cells (p < 0.01) (Figure 5b). This was accompanied by a significant decrease in branchpoints formed by CLIC1 knockdown (p < 0.001) (Figure 5c). The decrease in surface area coverage in CLIC1 knockdown could have been due to the previously established proliferative defect in CLIC1 knockdown HUVEC, however the reduction in branchpoint formation was novel, indicating that reduced CLIC1 expression inhibits endothelial network formation and branching.

### Reduced CLIC1 expression affects capillary-like sprouting and branching

Next, we assessed the effect of CLIC1 knockdown on capillary-like tube formation in a fibrin bead assay, which allowed for assessment of endothelial growth, sprouting, branching, and lumen formation. For this assay, CLIC1 knockdown and control HUVEC were attached to dextran-coated beads and embedded in a fibrin clot with fibroblasts seeded as a monolayer on top of the clot. The clot was cultured in EGM-2 medium, and HUVEC morphogenesis was monitored and photographically documented for 11 days. As previously reported, resultant sprouts from this assay are multicellular, lumen-containing processes [31,37]. In this assay, sprouting is noticeable as early as three days post embedding. Sprouts are reported to extend, anastomose, and undergo tubulogenesis from day 4 until the termination of the assay at day 11.

**Figure 6:**
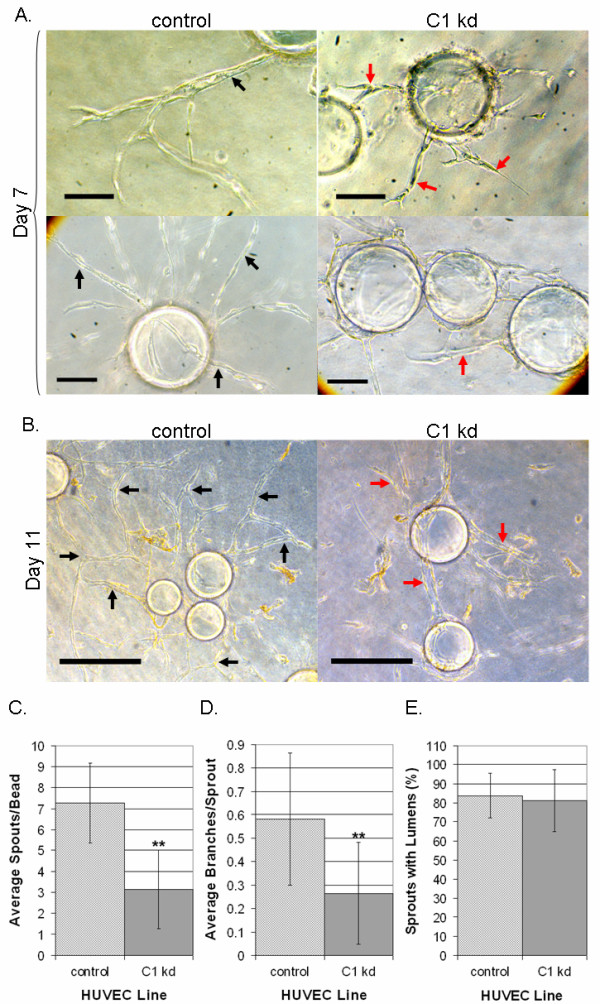
**Reduced CLIC1 expression affects capillary-like sprouting and branching**. A. Microscopy on day 7 of the fibrin bead assay showed that endothelial cells with CLIC1 knockdown had altered sprouting morphology. CLIC1 knockdown sprouts tended to be shorter, less branched, and less robust. Black arrows indicate long, robust, lumen-containing sprouts while red arrows indicate stunted tube structures. Scale bar, 10 μm. B. Microscopy on day 11 of the fibrin bead assay showed that endothelial cells with reduced CLIC1 expression formed tube-like structures; however these structures were stunted and possessed a branching defect when compared to structures formed by control cells. Black arrows indicate anastomosis or long tube-like structures. Red arrows indicate lack of branching or stunted tube-like structures. Scale bar, 30 μm. C. The average number of sprouts formed per bead was tabulated and quantified, shown here with standard deviation. Reduced CLIC1 expression was found to be accompanied by a significant reduction in the average number of sprouts per bead. D. Quantification of the average number of branches per sprout indicated that CLIC1 knockdown endothelial cells formed significantly fewer branches per sprout. The average number of branches per sprout is shown with standard deviation. E. Quantification of the number of lumen-containing sprouts revealed no significant difference between control cells and cells with CLIC1 knockdown. The average percentage of lumen-containing sprouts relative to total number of sprouts per cell line is shown with standard deviation. (**p < 0.001).

Our results indicated that knocking down CLIC1 expression resulted in stunted capillary sprouting and branching with little apparent effect on lumen formation (Figure 6a-b). Upon quantification of results from day 11, we found that the number of sprouts per bead and the average number of branches per sprout were significantly decreased in the CLIC1 knockdown group (p < 0.001) while the number of lumen-containing sprouts was not different (Figure 6c-e). The branching defect found in this assay was consistent with the branching defect found in the previous capillary-like network formation assay. Together, these data demonstrate that CLIC1 expression is required for capillary sprouting and branching morphogenesis, when assessed *in vitro.*

## Discussion

We have previously shown that CLIC4 knockdown results in lower endothelial cell growth and inhibited network formation, similar to the CLIC1 knockdown results shown here [14]. However, in contrast to the failure of CLIC4 knockdown cells to form networks, CLIC1 knockdown cells formed rudimentary networks but had a branching morphogenesis defect whereby cell aggregates formed at branchpoints. We suspect these cell aggregates were a manifestation of the migratory defect and a failure of knockdown cells to undergo appropriate branching morphogenesis. This would be consistent with the observation that CLIC1 knockdown reduced endothelial cell migration, whereas CLIC4 knockdown has no effect on migration. Another difference our comparison highlights is the lack of a lumen formation defect in CLIC1 knockdown endothelial cells in contrast to the lumen formation defect previously found with CLIC4 knockdown [14]. To explore the possibility of functional redundancy between CLIC1 and CLIC4, we made several attempts to generate HUVEC lines with both CLIC1 and CLIC4 knockdown. In contrast to HUVEC introduced with two different control vectors, all attempts to create double knockdown cell lines did not result in viable HUVEC. We found this observation consistent with the hypothesis that CLIC1 and CLIC4 may possess functional redundancies.

CLIC1 is found to be significantly up-regulated in highly metastatic gallbladder carcinoma cell lines [38]. More generally, chloride transport is reported to be integral in generating electrical signals that guide cell migration to wounds in corneal epithelium [39], and chloride channels are implicated directly in enabling glioma migration as well as regulating breast cancer invasiveness [40,41]. One may thus hypothesize that CLIC1 is required for endothelial cell motility based upon its potential function as an ion channel.

Extensive work has been done to validate CLIC1 as an anion channel that can auto-insert into artificial bilayers and produce conductance [5,22], however the channel selectivity is found to be poor with conductance being based on anion concentration [1,4,21,22,42]. As an anion channel, CLIC1 is redox-regulated and its sequence contains the putative transmembrane domain (PTM) purported to be essential for its proper integral membrane channel characteristics [21,43]. CLIC1 ion channel activity is also shown to be pH-dependent with activity lowest around neutral pH [22]. Structural experiments show that low pH may stimulate the PTM for insertion [11,44,45]. It will be important to assess whether these mechanistic features of CLIC1 are important for endothelial cell motility.

One of the most interesting structural characteristics of CLIC1 is the fact that it can exist as both an integral membrane protein and a soluble cytoplasmic protein. In contrast to the well-documented anion channel activity of CLIC1, the roles of CLIC1 independent of its channel activity are largely unknown. Studies demonstrate that a variety of CLIC proteins interact with the actin cytoskeleton either directly [9,46,47] or indirectly mediated by scaffolding proteins [27,48,49]. With this in mind, we postulate that CLIC1 may be regulating endothelial cell migration by regulation of cytoskeletal elements.

Endothelial cell migration and adhesion also depend on appropriate integrin expression [50]. Studies show that adhesion to the extracellular matrix through integrin heterodimers is essential for proper endothelial cell motility, and endothelial migration is at its greatest with intermediate levels of adhesion [51,52]. Of interest to our study are integrins αVβ3, which can bind with fibronectin; αVβ5, which binds only vitronectin; integrin subunits α2 and β1, which are known for binding collagens, laminins, and possibly fibronectin [53]; and the integrin α3 subunit, which binds fibronectin [54]. We found that reducing CLIC1 expression increased β1, α3, and αVβ3 expression while decreasing αVβ5 expression (Figure 3), suggesting a role for CLIC1 in mediating integrin presentation and a means by which CLIC1 may be affecting endothelial migration. By increasing the surface expression of β1, α3, and αVβ3, CLIC1 may be increasing endothelial cell adhesion to the extracellular matrix, inhibiting motility by preventing the cell from breaking its contact with the extracellular matrix. These shifts in integrin expression also provide a possible explanation for the cell growth and viability defects [55]. In addition, it is possible that CLIC1 alters integrin binding affinity for their ligands through inside-out signaling resulting in increased integrin expression but less efficient ligand binding [56]. To determine a mechanism by which CLIC1 may be influencing integrin cell surface expression, we conducted Western blotting for β1 and β5 integrin subunits and found that the protein levels of these integrins were unchanged by CLIC1 knockdown (data not shown). Based on these preliminary results, we hypothesize that changes to integrin cell surface expression are a result of altered cell trafficking as integrins cycle through the endosomal pathway and as a chloride channel, CLIC1 contributes to endosomal acidification [57].

In summary, we demonstrated here that CLIC1 is involved in several steps of angiogenesis *in vitro *and concluded that CLIC1 plays a role in mediating endothelial cell growth, branching morphogenesis, and migration, possibly via regulation of integrin expression. We found that the CLIC1 and CLIC4 knockdown phenotypes are similar in that both result in reduced cell growth, modestly increased cell viability, and inhibited network formation, but are different in that only CLIC1 knockdown inhibits migration and only CLIC4 knockdown affects lumen formation. It will be important to understand the molecular mechanisms by which CLIC1 functions in these diverse endothelial cell behaviors.

## Competing interests

The authors declare that they have no competing interests.

## Authors' contributions

JJT performed all experiments and analyses. JJT and JK wrote the manuscript. JK provided oversight for the research. All authors read and approved the final manuscript.
